# Dorsolateral Prefrontal Activation in Emotional Autobiographical Task in Depressed and Anxious College Students: An fNIRS Study

**DOI:** 10.3390/ijerph192114335

**Published:** 2022-11-02

**Authors:** Yan Zhang, Xiaoqin Li, Ying Guo, Zhe Zhang, Fang Xu, Nian Xiang, Min Qiu, Qiang Xiao, Pu Wang, Hui Shi

**Affiliations:** 1 School of Educational Science, Huazhong University of Science and Technology, Luoyu Road No. 1037, Hongshan, Wuhan 430074, China; 2 School of Psychology, Sichuan Normal University, Chengdu 610021, China; 3 Humanities Department, Huazhong University of Science and Technology, Wuhan 430074, China; 4 Department of Neurology, Hospital of Huazhong University of Science and Technology, Wuhan 430074, China; 5 Department of Rehabilitation Medicine, The Seventh Affiliated Hospital, Sun Yat-sen University, Shenzhen 518000, China; 6 Department of Clinical Psychology, Beijing Chao-Yang Hospital, Capital Medical University, Beijing 100020, China

**Keywords:** anxiety, depression, emotional autobiography, the dorsolateral prefrontal cortex, fNIRS

## Abstract

Objective: The dorsolateral prefrontal cortex (dlPFC) is strongly associated with mood symptoms. This study used functional near-infrared spectroscopy (fNIRS) technology to explore the features of brain neural activity in the dlPFC of anxious and depressed college students, during an emotional autobiographical memory task, and to understand the differences in brain cognitive mechanisms caused by anxiety and depression. Methods: A simple random sampling method was used to test 440 college students at a university with a healthy control group (HC, 220 participants), a pure depression group (PD, 92 participants), and a pure anxiety group (PA, 128 participants). The average oxyhemoglobin in the dlPFC of the subjects during the emotional autobiographical memory task was collected by a 53-channel functional near-infrared spectroscopy imaging device. Results: The activation of the left dlPFC (ch13) in the pure depression group was significantly higher than in the pure anxiety group. The activation of the right dlPFC (ch48) was significantly higher under positive emotions than under negative emotions. The interaction between emotion valence and group was marginally significant, and the activation of the right dlPFC (ch41) in the pure depression group was significantly higher under positive emotion than in negative emotion. The activation of the pure depression group under positive emotions was significantly higher than that of the pure anxiety group. In comparison, the activation of the pure depression group under negative emotions was significantly lower than that of the healthy control group. The results of correlation analysis showed that the activation of the left dlPFC (ch13) was significantly negatively correlated with anxiety in positive emotions, but the activation of the right dlPFC (ch34, ch42) was significantly positively correlated with anxiety in positive and negative emotions. Conclusions: The right dlPFC was insensitive to positive emotions in college students with high-anxiety symptoms, whereas this region was insensitive to negative emotions in college students with high depressive symptoms, which might be one of the critical differences in the cognitive mechanisms of anxiety and depression. Furthermore, left and right dlPFC activation correlated differently with anxiety. The higher the anxiety level, the lower the activation on the left side, and the higher the activation on the right side. The results suggested that anxiety might reduce the function of the left dlPFC.

## 1. Introduction

Different mental health problems have been experienced globally since the COVID-19 outbreak. Depression and anxiety are common affective disorders. According to scientific briefs published by the World Health Organization, the global prevalence of anxiety and depression increased substantially by 25% in the first year of the COVID-19 pandemic, with young adults being the most affected group. Studies have proven that depression and anxiety disorders can lead to different degrees of cognitive dysfunction and clinical symptoms, and neuroimaging studies have also shown that the two can lead to changes in cerebral cortex function, which seriously affects the quality of life [1].

Studies have proved that adults with a generalized anxiety disorder (GAD), or severe anxiety symptoms, have worse executive function performance than healthy groups [2]. Similar to anxiety, depressive symptoms are also associated with poorer performance on executive function tasks [3]. Patients with depressive disorder have cognitive deficits, manifested in weakening cognitive functions, such as verbal fluency, attention switching, and working memory. The development of brain imaging techniques has allowed the use of portable brain imaging techniques to detect functional activity patterns within the cerebral cortex. The most prominent of these is functional near-infrared spectroscopy (fNIRS) [4]. The spectroscopy offered by fNIRS can acquire real-time changes in the concentrations of oxyhemoglobin (oxy-Hb) and deoxyhemoglobin (deoxy-Hb), and dynamically monitor the physiological and pathological processes of the cerebral cortex. Furthermore, cerebral oxygen metabolism elevates when performing cognitive tasks with brain load. Cortical hemodynamic changes are captured by fNIRS to quantify blood oxygenation, which indirectly reflects brain function.

The dorsolateral prefrontal cortex (dlPFC) is strongly associated with mood symptoms. Liu et al. (2014) found that the severity of anxiety is related to the Oxy-Hb activation of the right lateral prefrontal cortex, and the severity of depression is associated with the Oxy-Hb activation of the left and right lateral prefrontal cortex and the medial dorsal prefrontal cortex [5]. These results suggest that oxy-Hb activation in the dlPFC can reflect the severity of clinical anxiety and depressive symptoms. Noda et al. also found that NIRS signal changes in the right dlPFC were negatively correlated with the severity of depression [6]. However, conclusions are inconsistent. Kameyama et al. found no correlation between NIRS signal and the severity of depression in patients with bipolar disorder [7].

Studies on the correlation between the severity of anxiety and oxy-Hb activation are also inconsistent. Ganella et al. found task-related brain activation patterns using fNIRS [8]. In tasks requiring emotion regulation, anxiety disorder patients had insufficient activation of the ventrolateral prefrontal cortex (vlPFC) and dlPFC, and a negative correlation was found between the severity of anxiety and oxy-Hb activation of the prefrontal cortex. Adolescents with anxiety symptoms also demonstrated dlPFC hypoactivity when completing a multi-source interference task (MSIT) compared with healthy controls [9]. Nishimura et al. found that oxy-Hb changes in the lower-left prefrontal cortex were significantly associated with the frequency of panic attacks [10]. However, Marumo et al. found no correlation between oxy-Hb changes and anxiety scores on the State–Trait Anxiety Inventory (STAI) in emotion-related tasks [11]. Therefore, based on the inconsistent results of previous studies, this study used fNIRS technology to re-explore the different activation patterns of the dlPFC between depression and anxiety in college students.

Anxiety and depression can also lead to impairments in memory, especially episodic memory. A study has proved that patients with depressive disorders have reduced specificity of recalled content during autobiographical memory retrieval [12]. Depressed individuals showed stronger memory discrimination ability to negative stimuli. For non-clinical samples, individuals with more severe depressive symptoms showed stronger recognition biases to negative stimuli. Furthermore, individuals with higher depressive symptoms had increased amygdala activity and decreased DG/CA3 activity when recognizing negative stimuli, compared with neutral stimuli [13]. Kheirbek et al. also found that anxiety symptoms were associated with impaired episodic memory dysfunction [14]. Other studies have shown that individuals with more severe anxiety symptoms have impaired memory for negative stimuli, a negativity memory bias, or augmented memory for negative materials [15].

Previous brain imaging studies on episodic memory showed that depressed individuals exhibit anomalous activity in the vlPFC and subgenual anterior cingulate cortex during the processing of negative emotional stimuli [16] and decreased prefrontal function in the recall of general autobiographical events [17]. An fMRI study explored the neural basis of autobiographical memory in depression, comparing the differences in neural responses to an autobiographical memory task in depressed patients and healthy controls [18]. The depressed group exhibited lower activation in the right IFG than the control group. Few previous studies have involved dlPFC, but dlPFC plays a key role in regulating emotions and cognitive function. Thus, this study used an emotional autobiographical memory task to explore the activation features of the dlPFC of depressed and anxious college students, to understand the differences in the brain’s cognitive mechanisms related to anxiety and depression.

## 2. Material and Methods

### 2.1. Participants

The patients we selected were university students from Huazhong University of Science and Technology Hospital. The patients with anxiety and depression in our study were 440 young students (188 female) with an average age of 21.35 ± 3.59 years, and diagnosed using DSM-5 by three senior psychiatrists. Before their fNIRS monitoring, all subjects were assessed with the Mini International Neuropsychiatric Interview (MINI-Chinese version) and the Hospital Anxiety and Depression Scale (HADS), and satisfied the criteria of the healthy control group (HC), the pure depression group (PD), and the pure anxiety group (PA). The 220 HC participants included 90 males and 130 females (mean age ± SD: 21.41 ± 3.59), the 92 PD participants consisted of 55 males and 37 females (mean age ± SD: 20.84 ± 2.83), and the 128 PA participants consisted of 67 males and 61 females (mean age ± SD: 21.58 ± 4.04).

All participants were right-handed and had normal hearing and normal, or corrected-to-normal, vision. All participants signed written informed consent voluntarily before participation in the study and were informed of relevant information about the research process. This study was approved by the Medical Ethics Committee of Huazhong University of Science and Technology.

### 2.2. Hospital Anxiety and Depression Scale (HADS)

The Hospital Anxiety and Depression Scale was developed by Zigmond and Snaith in 1983, mainly to screen for anxiety and depression [19]. The HADS scale contains 14 items, which can assess anxiety and depression. The 1st, 3rd, 5th, 7th, 9th, 11th, and 13th questions are anxiety items, and the 2nd, 4th, 6th, 8th, 10th, 12th, and 14th questions are depression items [20]. The anxiety and depression two subscale scores are 0–7, representing healthy psychological status, and 8–21, representing psychological distress [21]. A subscale score of ≤8 indicates a healthy psychological status, and a score of ≥8 indicates a possible anxiety disorder or depression [22]. All participants were divided into three groups according to the HADS score: the healthy control group, with HAD-A ≤ 7 and HAD-D ≤ 7, the pure depression group, with HAD-A ≤ 7 and HAD-D ≥ 8, and the pure anxiety group, with HAD-D ≤ 7 and HAD-A ≥ 8.

For the assessment of depression and anxiety, scales are still the main measuring tool in current research. The mainstream scales are the Hamilton Depression Rating Scale (HAM-D) for measuring depression and Hamilton Anxious Rating Scale (HAM-A) for measuring anxiety [23]. Although research continues to examine the construct validity of the scales to adequately discriminate between symptoms of depression and anxiety, the available findings show that different scales lead to different conclusions. A recent study confirmed that the Depression, Anxiety and Stress Scales (DASS-21) had difficulty in properly identifying and discriminating between symptoms associated with depression and anxiety [24], while the Multidimensional Anxiety Scale for Children (MASC) and the Center for Epidemiologic Studies-Depression Scale (CES-D) were successful in discriminating anxiety and depression [25]. However, among studies discriminating between depression and anxiety, researchers usually use HADS or HAM as a criterion to verify the validity of other scales to discriminate between depression and anxiety [26]. This has important implications for the feasibility of using HADS or HAM as a tool to discriminate anxiety and depression symptoms.

HAM was developed in the late 1950s and has been the standard measure of depression [27]. However, there is evidence that HAM has poor inter-rater reliability, retest reliability, and content validity [28]. However, self-assessment scales are more realistic, accurate and objective. Others-assessment scales may be more subjective, ignoring the subjective feelings of patients. Patients with depression, differing from other mental disorders, have certain self-cognition ability and can accurately assess their mental state. The HADS was a brief self-report scale designed for measuring the severity of depressive or anxious states [29] and it performs well in assessing the severity of depression and anxiety and has been proven to have good reliability and validity [30]. HADS has been translated into different language versions [31,32] and is widely used in adults [33], adolescents [34], and psychiatric populations [35]. Additionally, HADS was originally designed for depression screening of patients with somatic diseases. Later, the scale removed the items for measuring somatic symptoms. Since HADS does not include any items related to suicide and sexual symptoms, it is suitable for patients with moderate severity [36]. Therefore, we used the HADS to accurately measure depression and anxiety.

### 2.3. Emotional Autobiographical Memory Task (EAMT)

In the field of AM, studies mostly use Williams’ autobiographical memory task paradigm (AMT) [37] to measure autobiographical memory. AMT requires subjects to respond to a series of words, that is, to extract the autobiographical memory of the corresponding content according to the cue words. A large number of studies have improved the AMT paradigm. Cues are no longer limited to words but range from verbal cues, such as words, to non-verbal cues, such as music [38], odor [39], and images [40]. Studies have approved the effectiveness of pictures as cues for autobiographical memory. For example, Haj et al. found pictorial cues could elicit higher autobiographical memory than the no-cue condition in both patients with AD and cognitively normal older adults [41]. Anderson et al. used the AMT paradigm and visual pictures as cues, which proved the importance of visual image cues in direct retrieval and generative retrieval of autobiographical memory [42]. Therefore, in this study, we replaced the words with emotional pictures as cues.

Pictures in AMT were sourced from the Chinese Affective Picture System (CAPS) [43], which is a localized affective picture system in China. Pictures in CAPS have been proven to have a high internal consistency and test–retest reliability in valence, arousal, and dominance [43]. Three pictures were selected from CAPS that varied in terms of valence: positive, neutral, and negative. At the same time, fNIRS technology was used to record the participants’ brain activation while viewing emotional pictures.

**Figure 1:**
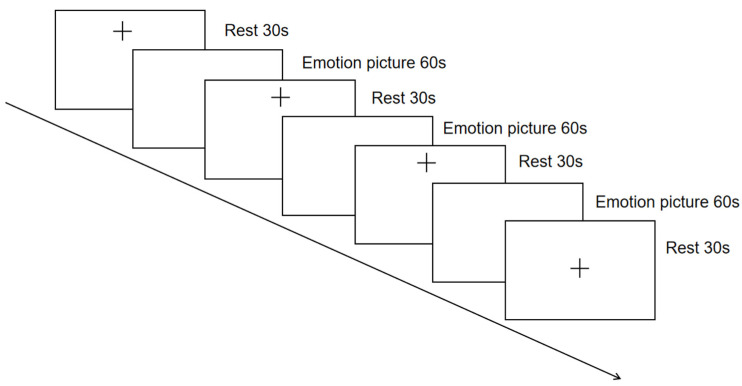
Emotional autobiographical memory task design.

The experimental task materials were presented to the participants through PowerPoint, and the presentation order of the three emotional pictures was randomized among the participants. Voice instructions were provided before the emotional pictures were presented, and the participants and the experimenter did not communicate during the task. EAMT prompts the participant to recall the events they have experienced according to each cue picture as quickly as possible, and the recalled events should be as specific and detailed as possible, involving events, places, and people. The duration of the EAMT was approximately 300 s, and the task design is shown in Figure 1, with a total of three stages. The first stage was a 30-s resting stage. The second stage had a total of three task periods, and the participants performed a 60-s recall according to the positive, negative, and neutral cue pictures. A 30-s break was given between each recall task, during which the subjects closed their eyes and rested. The third stage was a 30-s resting stage.

### 2.4. Functional NIRS Measurement

**Figure 2:**
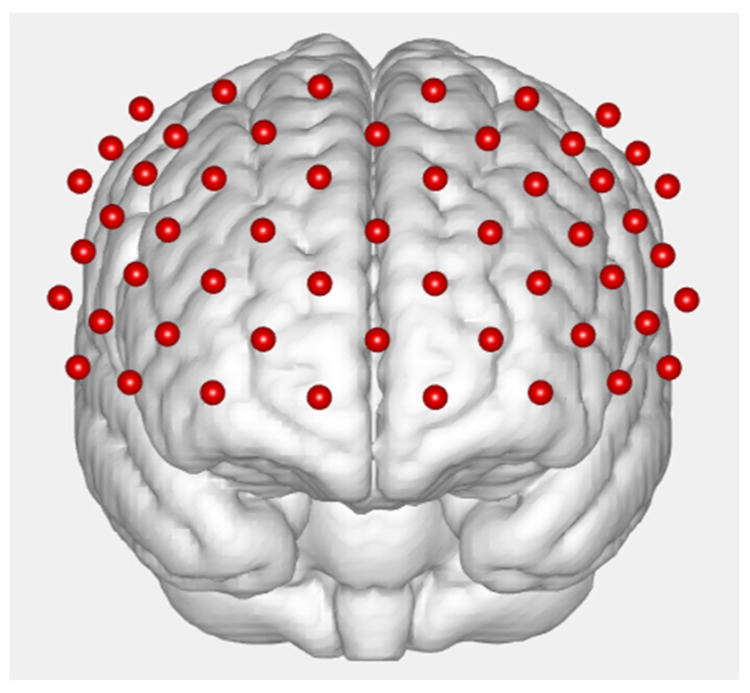
A schematic diagram of the distribution of the fNIRS photopoles and channels.

While performing the autobiographical emotion memory task, the subjects were tested by functional near-infrared spectroscopy. A 53-channel fNIRS machine (BS-7000, Wuhan Znion Technology Co., Ltd., Wuhan, China) was used to measure the relative concentration changes of oxygenated hemoglobin ([oxy-Hb]) using two wavelengths (690 and 830 nm) of infrared light, based on the modified Beer–Lambert law [44]. The sampling rate was 100 Hz. The measurement area between a detector and source probe pair was defined as a channel (ch). The source–detector probes were placed on the prefrontal areas (see Figure 2).

### 2.5. Data Statistics and Analysis

The fNIRS data were preprocessed by Matlab 2014a (Mathworks Co., Ltd., Natick, MA, USA) based Homer 2 (David Boas) software, and the HADS data and the average oxyhemoglobin (Avg-HbO) data were sorted and analyzed by IBM SPSS 22.0 (SPSS, Inc., Chicago, IL, USA).

The comparison of oxyhemoglobin in different groups at different emotional stages was performed by repeated-measures analysis of variance, and the correlation coefficient was used to describe the correlation between anxiety and depression and oxyhemoglobin. A value of *p* < 0.05 was considered statistically significant.

## 3. Results

### 3.1. Hospital Anxiety and Depression Scale (HADS) Scores

One-way ANOVA was used to test the differences in scores of anxiety and depression subscales among the three groups to verify the validity of the grouping. The results showed significant differences in the scores of the anxiety and depression subscales among healthy control, pure depression, and pure anxiety (Table 1).

### 3.2. Differential Analysis of Oxyhemoglobin Content in Anxiety and Depression under Different Emotions

**Figure 3:**
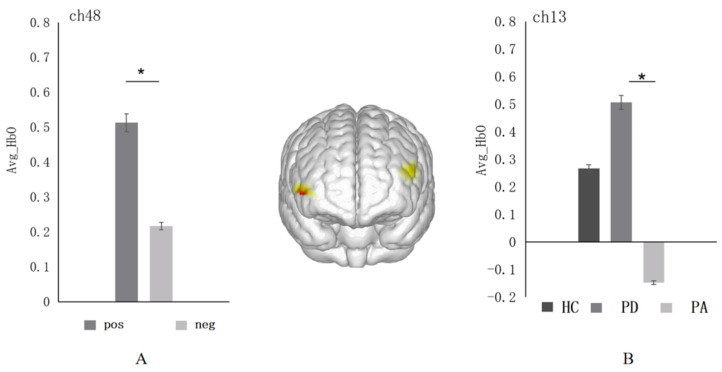
(**A**) Histogram of the distribution of the average oxyhemoglobin under different emotions in ch48 (right dlPFC) among three groups. (**B**) Distribution histogram of the average oxyhemoglobin in ch13 (left dlPFC) among three groups. Abbreviations: pos: positive emotion; neg: negative emotion; HC: the healthy control group; PA: the pure anxiety group; PD: the pure depression group. *: *p* < 0.05.

With the average oxyhemoglobin (Avg-HbO) as the dependent variable, 3 groups (HC/PD/PA) × 2 emotional valences (positive/negative) repeated measures analysis of variance were performed. First, a significant main effect between emotional valence was found in the right dlPFC (ch48) (*F* = 5.58, *p* = 0.02, *η*^2^*_p_* = 0.013, see Figure 3A). Post-hoc multiple comparisons found that this region was most sensitive to positive emotional pictures; that is, the Avg-HbO value evoked by positive emotions (*M* ± *SD* = 0.51 ± 0.11) was significantly higher than that of negative emotional pictures (*M* ± *SD* = 0.21 ± 0.11, *p* = 0.02). Next, a significant between-group main effect was found in the left dlPFC (ch13) (*F* = 2.83, *p* = 0.06, *η*^2^*_p_* = 0.013, see Figure 3B). Post-hoc multiple comparisons found that the Avg-HbO value induced by the pure depression group (*M* ± *SD* = 0.51 ± 0.22) in the emotional autobiographical task was significantly higher than that of the pure anxiety group (*M* ± *SD* = −0.15 ± 0.19, *p* = 0.02).

**Figure 4:**
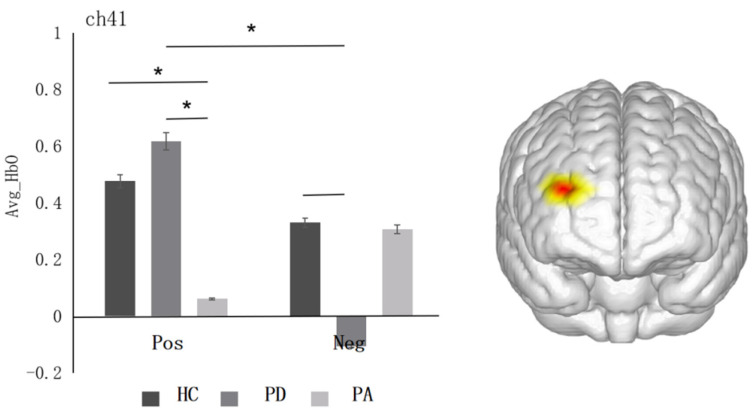
Histogram of the distribution of the average oxyhemoglobin in different emotions among three groups in ch41 (right dlPFC). *: *p* < 0.05.

The interaction between emotion valence and group was marginally significant in the right dlPFC (ch41) (*F* = 2.60, *p* = 0.076, *η*^2^*_p_* = 0.012, see Figure 4). Simple effects analysis revealed that, in the pure depression group, the activation level of positive emotions (*M* ± *SD* = 0.62 ± 0.20) was significantly higher than that of negative emotions (*M* ± *SD* = −0.11 ± 0.20, *p* = 0.03). In terms of the activation level of positive emotions, the pure depression group (*M* ± *SD* = 0.62 ± 0.20) was significant higher than the pure anxiety group (*M* ± *SD* = 0.06 ± 0.17, *p* = 0.03), and the healthy control group (*M* ± *SD* = 0.48 ± 0.13) was significantly higher than the pure anxiety group (*M* ± *SD* = 0.06 ± 0.17, *p* = 0.05). The activation level of negative emotions in the healthy control group (*M* ± *SD* = 0.33 ± 0.13) was marginally significantly higher than in the pure depression group (*M* ± *SD* = −0.12 ± 0.20, *p* = 0.06, marginally significant).

### 3.3. Correlation between the Severity of Anxiety and Depression and the Average Oxyhemoglobin

**Figure 5:**
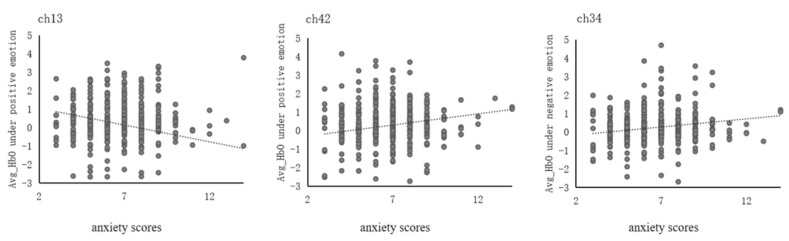
Correlation between the severity of anxiety and the average oxyhemoglobin under different emotions.

This study conducted a correlation analysis between Avg-HbO and anxiety and depression scores in positive and negative emotions, respectively, to explore the relationship between anxiety and depression severity and brain activation. The results were as follows (see Figure 5). In the left dlPFC, ch13 showed a significant negative correlation between Avg-HbO and anxiety scores under positive emotions (*r* = −0.01, *p* = 0.04). In the right dlPFC, ch42 showed a significant positive correlation between Avg-HbO and anxiety scores under positive emotions (*r* = 0.12, *p* = 0.01), and ch34 showed a significant positive correlation between Avg-HbO and anxiety scores under negative emotions (*r* = 0.11, *p* = 0.02). No correlation was found between Avg-HbO and depression scores under positive or negative emotions.

## 4. Discussion

This study used fNIRS technology to explore the activation features of the dlPFC of anxious and depressed college students, using the Medical Anxiety and Depression Questionnaire and the Emotional Autobiographical Task. Different activation features of the dlPFC were found. First, the right dlPFC of college students with high anxiety symptoms was insensitive to positive emotions, whereas this region of college students with high depressive symptoms was insensitive to negative emotions, which may be one of the important differences in the cognitive mechanisms caused by anxiety and depression. Furthermore, the correlation between the activation of the left and right dlPFC and the severity of anxiety was different. The results suggested that anxiety might reduce the function of the left dlPFC.

In this study, the severity of anxiety and depressive symptoms was quantified using the HADS score. Noda et al. found that the severity of depressive symptoms was inversely correlated with changes in NIRS signaling in the right dlPFC [6]. A study using PET in patients with depression found that metabolic changes in the dlPFC site were positively correlated with HAMD scale scores of depressive symptoms [45]. However, the present study did not find a correlation between the severity of depressive symptoms and the activation of dlPFC, which was consistent with the findings of Kameyama et al. They found no correlation between depressive symptom severity and NIRS signal in patients with bipolar disorder, probably because of the difference in the selection of participants [7]. Most of the participants selected in previous studies were patients with severe depression. However, studies have found that approximately 46% of patients with major depression have anxiety symptoms [46]; that is, anxiety and depression have a high comorbidity rate. Thus, this study selected pure depressed individuals and pure anxious individuals to explore the neural mechanism of the differences in cerebral hemodynamics between pure anxiety, pure depression, and healthy people. This study found that, in the left dlPFC, the average oxyhemoglobin in the pure depression group was higher than that in the healthy control group, which was inconsistent with previous findings. In previous studies, the activation of cerebral blood flow in healthy individuals was generally higher than in patients with depression [47]. The reason for the inconsistent results might have arisen from the fact that the tasks were different. The present study used an emotional autobiographical task and presented different emotional pictures to the subjects before the task began; thus, the task was different from the cognitive tasks commonly used in previous studies (such as verbal fluency tasks). The dlPFC is closely related to emotional symptoms, and the left dlPFC of depressed individuals shows an “overreaction” that exceeds that of healthy individuals when stimulated by emotional pictures. The results also suggested that this region might play an essential role in the emotion regulation process involved in the prefrontal lobe, and follow-up studies could pay more attention to the role of the left dlPFC in the study of the brain mechanism of depression.

Numerous studies have used the Verbal Fluency Task (VFT) to analyze the cerebral hemodynamic characteristics of patients with depression. On the one hand, the study results have shown that the hemodynamic response intensity of the forehead and bilateral temporal regions in patients with major depressive disorders is lower than that in the healthy control group during the VFT task [48]. On the other hand, a significant correlation was found between the severity of depressive symptoms and the activation of prefrontal subregions, but the correlation varied by depression type and brain region. For example, Liu et al. found that bipolar depressive patients and unipolar depressive patients had significant differences in the activation of the lateral prefrontal cortex and orbitofrontal cortex, and the left and right dlPFC were significantly different in patients with bipolar depression [5]. Activation of the lateral side was differentially correlated with the severity of depressive symptoms. Another study compared the relationship between brain activation and depression in patients under the verbal fluency task and the Tower of London task (TOL), which found that the functions of the dlPFC and vlPFC were healthier in patients with depression under the VFT and TOL. Impaired in controls, TOL activation levels in the inferior polar prefrontal cortex and left lateral prefrontal cortex were negatively correlated with the severity of depressive symptoms in depressive patients [49]. However, the impairment of episodic memory function caused by anxiety and depression has been confirmed by numerous studies [50,51]. Thus, the present study used the emotional autobiographical task for the first time, introduced emotional pictures of different valences, and explored the activation differences of the dlPFC of the brain under different emotions. Previous studies have confirmed that the activation of the prefrontal lobe under positive emotions is higher than that under negative emotions, indicating that individuals are more likely to perceive positive emotions. Although no correlation was found between the average oxyhemoglobin concentration in the dlPFC and depressive symptom scores, an important finding was that activation of the right dlPFC under positive emotions was significantly higher than that under negative emotions in the pure depression group. This finding also verifies the decline of prefrontal lobe function in processing negative materials in depressed individuals [16]. However, the pure anxiety group showed the opposite results to the pure depression group. The pure anxiety group showed higher activation of the right dlPFC under negative emotions than positive emotions, and the pure anxiety group showed a higher degree of activation to negative emotions. The pure depression showed a high degree of activation of positive emotions. Previous studies have shown that anxiety symptoms are associated with impaired memory for negative stimuli [52], whereas individuals with depressive symptoms show enhanced memory for negative stimuli [14]. Combined with the results of this study, depressed individuals did not need to invest more cognitive resources when faced with negative emotional pictures; thus, they also showed low levels of average oxyhemoglobin.

Regarding the relationship between the severity of anxiety symptoms and cerebral blood flow, previous studies have shown a significant correlation between the changes in oxyhemoglobin in the right anterior prefrontal cortex and the Temperament Personality Injury Avoidance Scale in healthy individuals [53]. Liu used fNIRS technology to find that the increase in oxyhemoglobin content of channel 21 located in the right dlPFC was significantly correlated with the severity of anxiety symptoms. The present study found differences in the association between the left and right dlPFC and anxiety symptom severity [5]. In the emotional task, the severity of anxiety symptoms under positive emotions was significantly positively correlated with the activation of the left dlPFC and significantly negatively correlated with the activation of the right dlPFC. The higher the anxiety level, the lower the activation level on the left side and the higher the activation level on the right side, suggesting that anxiety may reduce the function of the left dlPFC. However, the correlation coefficients in this study are small, possibly because the samples were non-clinically diagnosed individuals whose severities of anxiety and depression were not high.

The comorbidity of anxiety and depression has become one of the hot topics in the field of mental health and accurate discrimination between anxiety and depression is a difficult and challenging in current research [54]. Although researchers are trying to examine the construct validity of the scales to adequately discriminate between symptoms of depression and anxiety [55], the available findings show relatively little strong evidence supporting the discriminative validity of scales [24]. This is one of the limitations of this study. Therefore, future research is necessary to develop more detailed criteria for differentiation of anxiety and depression symptoms, and to further explore the change in brain activation in patients with different severities of different anxiety and depression symptoms.

## 5. Conclusions

This study used the fNIRS technique and the emotional autobiographical memory task to explore the features and differences in the activation of the dlPFC in pure anxiety, pure depression, and healthy individuals. The study found differences in the activation of the right dlPFC under different emotions in people with pure anxiety and pure depression, the correlation between the activation of the left and right dlPFC, and the severity of anxiety symptoms. The results of this study provide additional clues to effectively evaluate and further optimize the detection of anxiety and depression by fNIRS.

## Figures and Tables

**Table 1:** Differences in anxiety and depression scores among the three groups.

Sub-Scale	Group	*n*	*M*	*SD*	*df*	*F*	*p*
Anxiety	Healthy control	220	5.59	1.21	2	335.21	<0.001
	Pure depression	92	6.08	0.96			
	Pure anxiety	128	8.88	1.22			
depression	Healthy control	220	5.57	1.30	2	250.74	<0.001
	Pure depression	92	8.76	0.93			
	Pure anxiety	128	6.05	1.07			

## Data Availability

Data will be stored in a publicly accessible repository and will be available upon publication from the osf.io database (osf.io/saj3p).
